# Multicentre preclinical profiling of apramycin for the treatment of nontuberculous mycobacteria

**DOI:** 10.1016/j.ebiom.2025.106103

**Published:** 2025-12-31

**Authors:** Minh-Vu H. Nguyen, Michel Plattner, Deepshikha Verma, Ramya V. Krishnamurthy, Min Xie, Vinicius Calado Nogueira de Moura, Klara Haldimann, Ramon Lang, Katja Becker, Parvinder Kaur, Radha Krishan Shandil, Mayas Singh, Zackery P. Bulman, Thomas Dick, Shridhar Narayanan, Bettina Schulthess, Satoshi Mitarai, Charles L. Daley, Sven N. Hobbie

**Affiliations:** a Division of Mycobacterial and Respiratory Infections, Department of Medicine, National Jewish Health, Denver, CO, USA; b Division of Infectious Diseases, Department of Internal Medicine, University of California, Davis Health, Sacramento, CA, USA; c Institute of Medical Microbiology, University of Zurich, Zurich, Switzerland; d Colorado State University, Fort Collins, CO, USA; e Foundation for Neglected Disease Research, Bengaluru, India; f Center for Discovery and Innovation, Hackensack Meridian Health, Nutley, NJ, USA; g Department of Pharmacy Practice, Retzky College of Pharmacy, University of Illinois Chicago, Chicago, IL, USA; h National Reference Center for Mycobacteria, Zurich, Switzerland; i Research Institute of Tuberculosis, Kiyose-shi, Tokyo, Japan; j Department of Medicine, University of Colorado, Aurora, CO, USA; k Division of Clinical Bacteriology and Mycology, University Hospital Basel, Basel, Switzerland

**Keywords:** Chronic lung infection, Chemotherapy, Antibiotic, Apramycin

## Abstract

**Background:**

Current treatment regimens for nontuberculous mycobacteria (NTM) infections, especially *Mycobacterium abscessus*, have suboptimal clinical outcomes, demanding novel therapeutic options. The aminoglycoside apramycin (APR) has been suggested as a therapeutic alternative to amikacin (AMK). Here, we report experimental data as part of an ongoing preclinical assessment of APR.

**Methods:**

Antimicrobial activity against 828 isolates comprising the clinically most relevant NTM species were determined by broth microdilution. Bactericidal activity and killing kinetics of APR were determined across a variety of assay conditions against rough and smooth *M. abscessus* isolates *in vitro* and *in vivo*.

**Findings:**

Both the MIC_90_ and tentative epidemiological cutoff (TECOFF) of APR was 4 μg/mL for 358 *M. abscessus* isolates and thus eight-fold lower than the 32 μg/mL determined for AMK. For 25 *Mycobacterium chelonae* isolates, the MIC_90_ and TECOFF of APR was 4 μg/mL and thus four-fold lower than the 16 μg/mL determined for AMK. For 360 *Mycobacterium avium* complex (MAC) isolates the MIC_90_ was 32 μg/mL and the TECOFF was 64 μg/mL for both APR and AMK. The MICs of APR and AMK were largely indifferent to mucin, DNA, sputum or standard of care antimicrobials except bedaquiline, which appeared to exert an additive effect. 16 μg/mL of APR resulted in a multi-log CFU reduction of *M. abscessus* within 12 h, in contrast to AMK, which was bacteriostatic for at least 24 h up to a concentration of 256 μg/mL. This difference in bacterial killing was consistent across pH 6.0–7.4 in 7H9 and CAMHB culture medium, respectively. APR resulted in multi-log CFU reduction in surrogate caseum and, unlike AMK, demonstrated intracellular killing of *M. abscessus* and *M. avium* in macrophages. Concentrations of 16 μg/mL of APR or ≥128 μg/mL of AMK suppressed the dose-dependent *M. abscessus* frequency of spontaneous resistance to near below the detection limit. In a cystic fibrosis surrogate CFTR^−/−^ mouse infection model, APR demonstrated dose dependent CFU reduction of pulmonary *M. abscessus* 4530, by up to 2 logs at 64 mg/kg.

**Interpretation:**

For *M. abscessus* and *M. chelonae*, the APR MIC_90_ was four-to eight-fold lower than that of AMK, and demonstrated enhanced bacterial killing. Collectively, our findings suggest APR has potent activity against NTM and warrants consideration for further clinical development.

**Funding:**

This study was supported by the 10.13039/100000897Cystic Fibrosis Foundation (CFF) Therapeutic Development Award JUVABIS22W0 and CFF grant HOBBIE19I0.

**Research in context**

**Evidence before this study**

Infections with nontuberculous mycobacteria (NTM), most commonly caused by *Mycobacterium avium* complex (MAC) and *Mycobacterium abscessus,* is increasing globally and among the most challenging infectious diseases to treat. We searched PubMed for clinical trials, observational studies, guidelines, reviews in English on the treatment of “nontuberculous mycobacteria”, “*M. avium* complex”, or “*M. abscessus*” from January 1, 2000 through December 31, 2024. NTM pulmonary disease (-PD) is the most common clinical manifestation, necessitating prolonged multidrug antimicrobial regimens of at least 12 months after culture conversion and often complicated by considerable drug toxicity, frequent treatment failures, and high recurrence rates. Aminoglycosides, chiefly amikacin (AMK), are among the most potent antimicrobials against NTM but are associated with substantial risks of ototoxicity and nephrotoxicity, limiting their use to severe or challenging cases such as cavitary or macrolide-resistant MAC-PD and *M. abscessus* disease, the latter being notably difficult to eradicate. Thus, there is an urgent need for more effective and tolerable antimicrobials to treat NTM disease. Apramycin (APR), an aminoglycoside with a unique chemical structure and therefore predicted to have a more favourable toxicity profile than current aminoglycosides, has shown promise in an exploratory *in vitro* study of eleven *M. abscessus* isolates that demonstrated that APR is more potent and bactericidal than AMK against *M. abscessus*.

**Added value of this study**

Our study is a large multicentre preclinical profiling of APR against the most common nontuberculous mycobacteria (NTM) consisting of MAC, *M. abscessus*, *Mycobacterium fortuitum*, *Mycobacterium kansasii*, and *Mycobacterium chelonae*. Among the 828 NTM isolates collected from patients who resided in the USA, Switzerland, and Japan, the distribution of minimal inhibitory concentration (MIC) of APR, a measurement of *in vitro* potency, was eight-fold lower than that of AMK for *M. abscessus* and *M. chelonae* but was equivalent for *M. fortuitum*, MAC, and *M. kansasii*. Compared with AMK, APR killed *M. abscessus* more rapidly, better penetrated macrophages and killed more intracellular *M. abscessus,* and incurred spontaneous resistance less frequently. Further, APR potency was not compromised by sputum or caseum surrogate *in vitro* and translated into dose-dependent efficacy in a cystic fibrosis mouse model experiment.

**Implications of all the available evidence**

This study contributes a comprehensive preclinical evaluation of APR against a large collection of NTM isolates, including MAC and *M. abscessus*. Our findings corroborate prior *in vitro* data demonstrating that APR is more potent against *M. abscessus* than AMK. Further, we provide new evidence that APR has superior activity to AMK against *M. chelonae* and at least equivalent potency to AMK against MAC, *M. fortuitum*, and *M. kansasii*. In addition, experiments with *M. abscessus* revealed that APR not only achieves more rapid bactericidal and intracellular activity than AMK but also demonstrates *in vivo* efficacy in a cystic fibrosis mouse model. Collectively, the current evidence supports further clinical investigation of APR as a promising treatment for NTM disease, especially those caused by *M. abscessus* and *M. chelonae*.

## Introduction

New and better antimicrobials against nontuberculous mycobacteria (NTM) are urgently needed. NTM diseases have increased in incidence and prevalence worldwide^1^ and carry significant morbidity and mortality, with one study reporting an associated all-cause mortality as high as 59%.^2^ Unfortunately, their treatment requires a 12-month-minimum multidrug regimen fraught with adverse effects and suboptimal outcomes.^3^ Aminoglycosides constitute one of the most important antimicrobial classes against NTM and are recommended in multidrug regimens against cavitary, severe, or macrolide-resistant NTM pulmonary disease because of their association with better outcomes.^3^, ^4^, ^5^, ^6^ Amikacin (AMK) is the most frequently used aminoglycoside for these infections,^3^ to which most rapidly growing mycobacteria (RGM) and slowly growing mycobacteria (SGM) are susceptible.^7^^,^^8^ However, as much as current aminoglycosides like AMK are needed for therapy, their merits come at the cost of relatively high rates of ototoxicity and nephrotoxicity.^9^

Apramycin (APR), a distinct aminoglycoside scaffold that has recently entered development for use in humans,^10^ has been suggested for assessment of its therapeutic potential against NTM disease.^11^^,^^12^ It has been proposed to be less toxic than other aminoglycosides because of its unique chemical structure^13^, ^14^, ^15^ and to achieve an adequate pharmacokinetics/pharmacodynamics (PK/PD) area-under-the-curve (AUC) over minimal inhibitory concentration (MIC) target in isolates with an MIC up to 8 or 16 μg/mL depending on the pathogen and the site of infection.^10^^,^^16^, ^17^, ^18^ A preliminary exploratory study has shown that APR is more potent and bactericidal than AMK against 11 selected *M. abscessus* isolates, largely due to its chemical structure allowing it to circumvent the mechanisms behind the limited AMK susceptibility of *M. abscessus*.^11^

Here, we aimed to provide a thorough preclinical profiling of APR in comparison to AMK by describing their MIC distributions for the most common NTM and defining tentative epidemiologic cutoffs (TECOFFs), by challenging *in vitro* activity in the presence of mucin or sputum, by further assessing their *in vitro* activity in planktonic and intracellular time-kill kinetic experiments, by comparing the frequencies of resistance, and by studying the therapeutic potential of APR against *M. abscessus* in a cystic fibrosis (CF) mouse model. Collectively, these experiments comprehensively evaluated the *in vitro* efficacy and therapeutic potential of APR versus AMK against a large and diverse sample of clinical NTM isolates.

## Methods

### Acquisition of isolates and susceptibility testing

828 clinical NTM isolates consisting of both RGM and SGM were used for *in vitro* susceptibility testing at three study sites: National Jewish Health (Denver, CO, USA; *n* = 254), University of Zurich (Zurich, Switzerland; *n* = 278), and Research Institute of Tuberculosis (Kiyose-shi, Tokyo, Japan; *n* = 296) (Supplementary Table S1).

All three sites conducted susceptibility testing using broth microdilution according to the Clinical and Laboratory Standards Institute (CLSI) documents M24, 3rd edition and M24S, 2nd edition.^19^^,^^20^ Site-specific experimental details are summarised in Supplementary Table S2. In brief, RGM were incubated at 30 °C for 5–7 days, and SGM at 37 °C for 14 days, except *Mycobacterium xenopi* at 42 °C. Bacterial suspensions were adjusted to a 0.5 McFarland standard and further diluted in cation-adjusted Mueller Hinton broth (CAMHB) without OADC for RGM and with 5% OADC for SGM. The suspensions were then added to microtiter plates with pharmaceutical-grade APR (EBL-1003, Juvabis, Switzerland), AMK, or tobramycin (TOB). RGM plates were incubated at 30 °C for 2–5 days and SGM plates for 7–14 days at 35–36 °C, except for *M. xenopi* at 42 °C. Growth was checked visually without indicator dyes. Control over incubation conditions was confirmed to be critical for MIC determinations (Supplementary Table S3). A subset of isolates was tested at all three sites to test for data reproducibility and variability. TECOFFs were determined using the EUCAST ECOFFinder v2.1 curve fitting function, which provided statistical 97.5% and 99.0% cutoffs of MIC distribution.^21^

### Planktonic time-kill experiments

Time-kill kinetics were determined in 50 mL CAMHB supplemented with 0.05% polysorbate and antimicrobials. Erlenmeyer flask cultures were agitated at 120 rpm, 35 ± 2 °C. Cell inocula were prepared from well dispersed serial subcultures in Middlebrook 7H9 broth, adjusted to an OD_600_ of 0.5, and diluted 1:1000 in CAMHB to result in a cell density of about 10^5^–10^6^ CFU/mL. At the specified time points, 100 μL aliquots were sampled from each culture and serially ten-fold diluted in physiologic saline for CFU enumeration by colony count on Luria Bertani (LB) agar plates. Another luminescence-based methodology not requiring CFU enumeration was applied by transforming *M. abscessus* ATCC 19977 with bacterial luciferase-encoding plasmid 26161 (Addgene).^22^ Bacteria were suspended to an OD_600_ of 0.025 in 7H9 media supplemented with antimicrobials and 100 μL aliquots dispensed into a white 96-well round-bottom plate and incubated in a Biotek Cytation 5 imaging reader at 35 ± 2 °C with an hourly agitation period of 45s. The pH was adjusted and buffered with 2-morpholinoethanesulfonic acid (MES) as described previously.^14^

A surrogate caseum matrix was generated from cultured THP-1 cells (ATCC TIB-202, RRID: CVCL_0006) as described previously.^23^^,^^24^ *M. abscessus* exponential cultures grown in Middlebrook 7H9 broth (Sigma Aldrich) to an OD_600_ of 0.6–0.9 were spun down and resuspended in water to an OD_600_ of 0.7, resulting in a starting CFU/mL of approximately 10^8^. The bacterial suspension was added to the caseum surrogate in a 2:1 ratio (vol/wt), briefly homogenised with 1.4-mm zirconia beads, divided evenly into 1.5-mL microcentrifuge tubes, and incubated as standing cultures at 37 °C. At day 5, after the cultures entered stationary phase, 50 μL aliquots of bacterial culture in caseum surrogate were exposed to 0, 4, 16, 32, 64, or 256 μM of APR or AMK for 5 days, after which CFU was enumerated. Drug concentrations in μM were converted to μg/mL for consistent units in data plotting across experiments.

### Intracellular time-kill experiments

Intracellular killing was essentially determined as described previously for *Mycobacterium tuberculosis*.^25^ In brief, overnight cultures of THP-1 cells (ATCC TIB-202, RRID: CVCL_0006) were used to induce macrophage differentiation by 50 nM phorbol 12-myristate 13-acetate (PMA) for 48–72 h. THP-1 macrophages were infected with either *M. abscessus* ATCC 19977 or *M. avium* ATCC 19698 and treated for 3 and 7 days, respectively, with APR or AMK followed by cell lysis with 0.05% SDS and CFU enumeration on 7H11 agar plates. Uninfected cells were treated for 7 days with APR and AMK as a control for any potential drug-induced effects on the cells. The assay was performed as biological triplicates. A more detailed description of the methodology is presented as Supplementary Material.

### Frequency of resistance experiments

*M. abscessus* starter cultures were grown in Middlebrook 7H9 medium for 2–3 days, then harvested during the log phase at an OD_600_ of 0.4–0.8. The cell pellet was resuspended to an OD_600_ of 1.0, which was about 10^9^ cells/mL. Three 100 μL samples (around 10^8^ cells each) were plated onto three LB agar plates with the desired antimicrobial concentration. For CFU enumeration, the cells were diluted ten-fold in 7H9 medium to a density of about 10^4^, 10^3^, and 10^2^ cells/mL. Three 100 μL samples of the highest dilution were spread on three non-selective LB agar plates and incubated for 5 day at 37 °C. Resistance frequency was calculated by dividing the number of colonies on antimicrobial plates by the number on nonselective plates, adjusted for dilution.

### Sputum inhibition experiments

Sputum inhibition experiments were adapted from a method described previously.^26^ In brief, equal volumes of fresh sputum and antimicrobial working solution were mixed and incubated at 37 °C for 15 min prior to dialysing 100 μL of the mixture at 37 °C for 4 h against 1.8 mL of CAMHB in a Pierce 96-well microdialysis plate with a molecular weight cutoff of 3.5 kDa (Thermo Fisher Scientific). Mixing the antimicrobials with physiological saline, or 12% mucin, 8 mg/mL calf thymus DNA as sputum surrogate instead of sputum served as reference controls. The CAMHB dialysate was then serially two-fold diluted to determine the highest dilution that inhibits the growth of indicator *Escherichia coli* strains DH5α (wt) and DH5α-*rmtC* described previously.^27^ The fraction unbound was assumed to be 100% in the saline control, to infer an APR MIC of 1 μg/mL for both DH5α and DH5α-*rmtC*. For AMK and TOB, the reference MICs were determined as 1 and as 0.25 μg/mL, respectively, for DH5α; and as >128 μg/mL for both drugs against DH5α-*rmtC*. Changes in bacterial inhibition by dialysate were expressed as fold-MIC relative to the saline control.

### Mouse infection model

The mouse infection model used in this study has been described previously.^28^ In brief, surrogate CF, B6CFTR^tm1UNC^/CFTR^tm1UNC^ (CFTR^−/−^) mice characterised by impaired mucociliary clearance were bred at the Laboratory Animal Resources breeder facility of the Colorado State University (CSU). Female 6–8 weeks old mice were rested one week before intratracheal injection of 0.5 to 1 × 10^6^ CFU of *M. abscessus* subsp. *abscessus* strain 4530 (NR-44274) per mouse. At the time of study conduct, the *M. abscessus* mouse infection model had only been established for female CFTR^−/−^ mice, probably because male mice are more susceptible to mycobacterial infections and exhibit a higher mortality in response to infection, making it more challenging for male mice to survive extended study periods while maintaining a sustainable infection. The CFU count in both bacterial stocks and the inoculum were controlled by spreading duplicate serial dilutions onto 7H11-OADC agar plates followed by five days of incubation. Bacterial burden at the start of treatment was determined two days post infection. Treatment groups with dose levels ranging from 4 to 256 mg/kg BID of subcutaneous APR or AMK for eight days are detailed in Supplementary Table S4. Treated and vehicle control mice were sacrificed two days after the last drug administration to spread serially diluted lung, spleen, and liver homogenates onto 7H11-OADC agar medium for CFU counts. A more detailed description of the methodology is presented as Supplementary Material.

### Statistics

The statistical significance of differences in log CFU bacterial counts between start and end of treatments (*n* = 5 animals per group) was calculated by the GraphPad Prism software version 8.4.3 using one-way ANOVA followed by Dunnett's Multiple Comparison Test. Normality assumption has been confirmed by linearity in Q–Q plotting. Sample size was guided by precedent studies of the specific animal model reported in the literature, attempting to minimise the number of animals used while providing acceptable statistical power. Individual mice were randomly allocated to each group in a non-blinded fashion.

### Ethics

The National Jewish Health IRB conferred the antimicrobial susceptibility testing of human clinical isolates a non-human subjects determination, not requiring written informed consent (HS-3939). Sputum sample donations were received fully anonymised. Animal study procedures were performed in compliance with animal use guidelines (ARRIVE), reviewed and approved by the CSU Animal Care and Use Committee, licence 1474.

### Role of funders

The funding sources had no role in the study design, data collection, data analyses, data interpretation, or decision to publish.

## Results

### MICs and TECOFFs

**Fig. 1:**
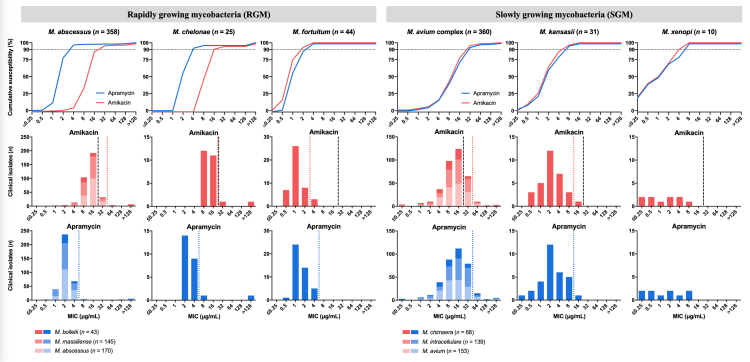
**Cumulative susceptibility and minimal inhibitory concentration (MIC) distributions of apramycin (APR) and amikacin (AMK) for the rapidly growing mycobacteria (RGM) *M. abscessus, M. chelonae,* and *M. fortuitum* and for the slowly growing mycobacteria (SGM) *M. avium* complex*, M. kansasii,* and *M. xenopi*.** The *M. abscessus* species is comprised of the three subspecies *M. abscessus, M. massiliense, and M. bolletii*. The *M. avium* complex is comprised of ten species including *M. avium* and *M. intracellulare* ssp *intracellulare* and ssp *chimaera*. In the cumulative plotting, the MIC_90_ cut-off is indicated by a horizontal dotted line. In the distribution bar charts, the tentative epidemiological cutoffs are indicated by a coloured dotted line but were not determined for *M. xenopi* due to small sample size. The black dashed vertical lines indicate the Clinical and Laboratory Standards Institute breakpoints for AMK (M24S, 2nd edition).

A total of 828 clinical NTM isolates were tested across three participating reference laboratories in the USA, Japan, and Switzerland. The composition of the RGM (*n* = 427) and SGM (*n* = 401) isolate panels at the species and subspecies level is detailed in Supplementary Table S1. The MIC distributions of APR and AMK for these NTM isolates are plotted in Fig. 1 and the MICs and TECOFFs are summarised in Table 1.

**Table 1:** Summary of susceptibility testing results including statistical TECOFF values.

Organism	Drug	*n*	MIC (μg/mL)			MIC_50_	MIC_90_	TECOFF^a^	
			Lowest	Modal	Highest			97.5%	99%
*M. abscessus*^b^	AMK	358	0.5	16	>128	16	32	32	32
	APR	358	0.5	2	>128	2	4	4	4
*M. chelonae*	AMK	25	8	8	>128	16	16	16	16
	APR	25	2	2	>128	2	4	4	4
*M. fortuitum*	AMK	44	0.5	1	4	1	2	2	2
	APR	44	0.5	1	4	1	4	2	2
MAC	AMK	360	0.25	16	>128	16	32	64	64
	APR	360	0.25	16	>128	16	32	64	128
*M. kansasii*	AMK	31	0.5	2	16	2	8	8	16
	APR	31	0.25	2	16	2	8	16	16

MIC, minimal inhibitory concentration; MIC_50_, concentration that inhibited 50% of isolates; MIC_90_, concentration that inhibited 90% of isolates; MAC, *Mycobacterium avium* complex; AMK, amikacin; APR, apramycin.

a TECOFF, tentative epidemiological cutoff. Statistical 97.5% and 99.0% curve fitting TECOFFs were determined using the EUCAST ECOFFinder v2.1.

b *M. abscessus* isolates comprised all three subspecies. Isolate details are provided in Supplementary Table S1.

The APR MIC was four-to eightfold lower than that of AMK for all *M. abscessus* isolates tested (Fig. 1, Table 1), including both smooth and rough morphotypes (Supplementary Fig. S1). The APR MIC for *M. chelonae* isolates was likewise four-to eightfold lower than that of AMK (Fig. 1) and about two-fold lower than that of TOB (Supplementary Fig. S2), which is considered to be the aminoglycoside of choice for treatment of *M. chelonae*.^6^^,^^29^ In contrast, APR MICs were generally equivalent to those of AMK for *M. fortuitum* and SGM (Fig. 1, Table 1). To not only compare MICs at the population level but also for individual isolates, we plotted the MIC of APR against that of AMK (Supplementary Fig. S3), indicating that isolates of lower susceptibility to the aminoglycoside AMK tended to be less susceptible to the aminoglycoside APR as well. For a subset of 273 NTM isolates, we also determined the susceptibility profile to other standard of care drugs as a reference (Supplementary Fig. S4).

The *in vitro* activity of APR was not compromised by mucin, DNA, or sputum (Supplementary Fig. S5). Checkerboard assays with APR and AMK in different *in vitro* conditions suggested no synergistic or antagonistic effects when combined with clarithromycin, clofazimine, imipenem, bedaquiline, or tigecycline (Supplementary Fig. S6). Combination with bedaquiline was interpreted as a possible additive effect.

### Frequency of resistance

**Fig. 2:**
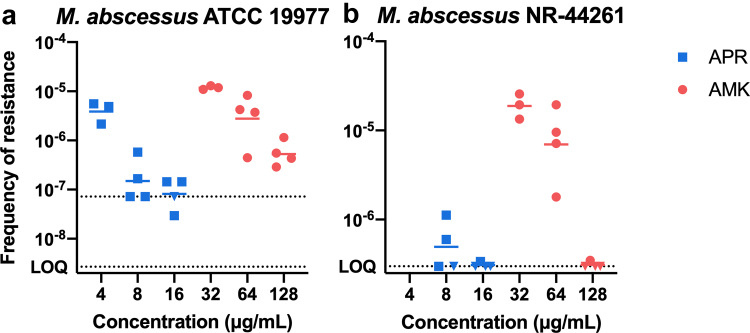
**Frequency of mutational *M. abscessus* resistance (FoR) to apramycin (APR) and amikacin (AMK).** (a) *M. abscessus* ATCC 19977. (b) *M. abscessus* NR-44261. All studies were performed on selective LB agar plates incubated at 37 °C for 5 days. Data are represented as scatter plot, the centre bars represent the geometric mean (*n* = 3–4 technical replicates per strain and drug concentration tested). The lower limits of quantification (LoQ) defined by ≤ 1 CFU per plate are indicated by dotted lines. Circular symbols at the LoQ are equivalent to one resistant CFU, triangular symbols pointing downward indicate the absence of resistant CFUs and thus a frequency below that LoQ.

The frequency of spontaneous resistance of *M. abscessus* at 37 °C was below 10^−6^/CFU for growth on agar plates containing ≥8 μg/mL of APR. In contrast, and probably in accordance with the higher MIC of susceptible strains when compared to APR, the frequency of spontaneous AMK resistance at 37 °C was approximately 10^−5^/CFU for growth on 32 μg/mL of AMK and below 10^−5^/CFU for growth on >32 μg/mL of AMK (Fig. 2).

### Planktonic and intracellular time-kill kinetics

**Fig. 3:**
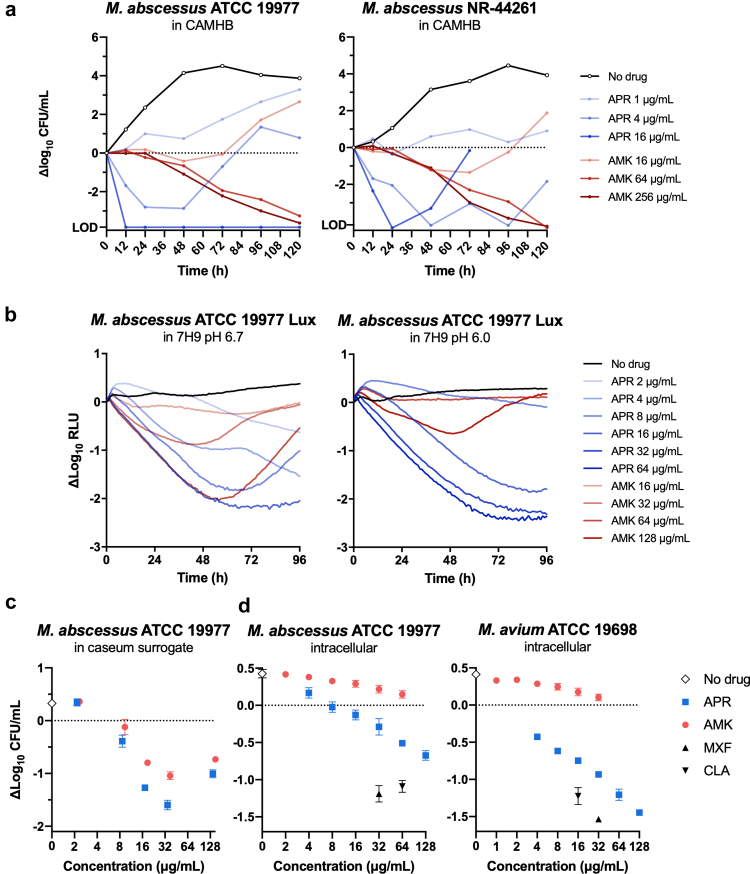
**Dose-dependent killing of planktonic and intracellular NTMs by apramycin (APR) in comparison to amikacin (AMK).** (a) Time- and dose-dependent CFU reduction of *M. abscessus* reference strains ATCC 19977 (smooth) and NR-44261 (rough) in CAMHB (pH 7.4) determined by sampling, plating, and CFU counting at different time points over 120 h. (b) Luminescence based killing kinetics in standard Middlebrook 7H9 (pH 6.7) and in a 7H9 medium that has been buffered with MES to pH 6.0. (c) Dose–response kill curve for stationary-phase *M. abscessus* ATCC 19977 in caseum surrogate following five days of drug exposure (mean ± SD; *n* = 3 technical replicates). (d) Dose response curve of intracellular killing of *M. abscessus* ATCC 19977 by day 3 and of *M. avium* ATCC 19698 by day 7 (mean ± SD; *n* = 3 biological replicates). MXF, moxifloxacin; CLA, clarithromycin.

Following the determination of MICs as a measure of bacterial growth inhibition by APR and AMK, we also studied their bactericidal activity over time against two *M. abscessus* reference strains in a time-kill kinetics assay. In CAMHB, at a physiological pH of 7.4, an APR concentration of 1 μg/mL, corresponding to 2 × the MIC, inhibited bacterial growth but did not result in a notable net CFU reduction. APR concentrations of 4 and 16 μg/mL, however, resulted in greater than 2- and 3-log CFU reductions within 24 h, respectively (Fig. 3a). AMK concentrations of up to 256 μg/mL, corresponding to 128 × the MIC, did not result in any notable CFU reduction within the first 24 h. The number of viable cells started to decrease gradually over time at incubation times ≥48 h in response to AMK concentrations ≥64 μg/mL (32 × MIC).

To study the effect of a slightly acidic environment on the bactericidal potency of APR, we performed a similar assay with an *M. abscessus* strain expressing a luciferase reporter, using ATP-dependent luminescence as a surrogate for cell viability. In regular 7H9 medium with a pH of 6.7, APR concentrations ≥8 μg/mL resulted in a reduction of luminescence of at least 2 logs after 48 h (Fig. 3b). An AMK concentration of 64 μg/mL reduced the luminescence with a kinetic comparable to that of 16 μg/mL of APR, but with luminescence increasing again after 60 h. In MES buffered 7H9 medium with a more acidic pH of 6.0, four-fold higher APR concentrations of ≥32 μg/mL were required to achieve a similar effect as ≥8 μg/mL did at pH 6.7 (Fig. 3b). An APR concentration of 16 μg/mL reduced the luminescence only by 1-log within 48 h, and a concentration of 8 μg/mL did not result in any notable reduction in luminescence. AMK concentrations of up to 128 μg/mL did not result in a considerable reduction in luminescence of cells incubated at pH 6.0.

Caseum, a necrotic matrix component in mycobacterial lung lesions, has been described as a reservoir of drug-recalcitrant persisting mycobacteria. APR demonstrated effective killing of stationary *M. abscessus* in caseum surrogate resulting in a higher CFU reduction when compared to AMK (Fig. 3c).

Next, we studied the effect of APR and AMK against intracellular NTM in THP-1 macrophages in RPMI medium. Microscopic evaluation of cell layer integrity and cell morphology did not show any drug-induced effects during 7 days of incubation when compared to untreated control. APR was found to kill intracellular *M. abscessus* ATCC 19977 (E_max_ = −0.67 log CFU/mL) and intracellular *M. avium* ATCC 19698 (E_max_ = −1.45 log CFU/mL) in a dose-dependent manner, with the *M. avium* reference strain (MIC = 4 μg/mL at 37 °C) appearing to be more susceptible to intracellular APR killing than the *M. abscessus* reference strain (MIC = 0.5 μg/mL at 37 °C) (Fig. 3d). Intracellular activity of APR appeared to be lower than that of clarithromycin and moxifloxacin, whereas AMK was not even bacteriostatic at 64 μg/mL, the highest concentration tested. The dose-dependent kinetic of intracellular CFU reduction over time is additionally provided as Supplementary Fig. S7.

### Efficacy in animal infection models

**Fig. 4:**
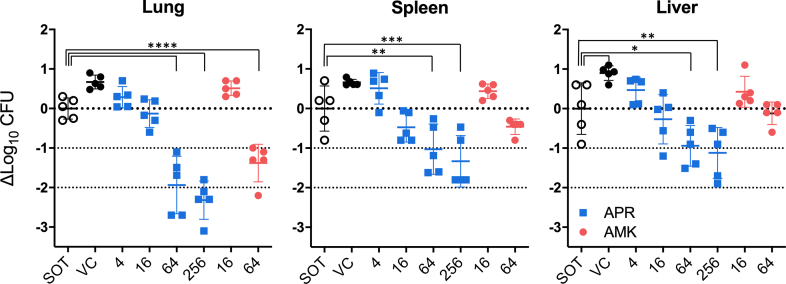
***In vivo* efficacy of apramycin (APR) against *M. abscessus* in a CFTR^−/−^ mouse lung infection model in comparison to amikacin (AMK).** Two days post intratracheal infection with 0.5–1 × 10^6^ CFU of *M. abscessus* 4530, the bacterial burden in lung, spleen and liver was determined as 5.3, 4.8, and 4.9 log CFU/g, respectively, prior to start of treatment (SOT). Mice were treated with plasmalyte vehicle (VC), 4, 16, 64, or 256 mg/kg APR, or 16, 64 mg/kg AMK s.c. BID for eight consecutive days. Two days after the last dose, the bacterial burden was again assessed by plating serial dilutions of organ homogenates on nutrient 7H11 agar, and CFU counts after incubation for 11 days at 32 °C. Data are represented as scatter plot, the centre bars represent the mean ± SD as error bars (*n* = 5 animals per group). Statistical significance of the difference in log CFU bacterial counts between start and end of treatment was calculated by using the one-way ANOVA followed by Dunnett's Multiple Comparison Test (∗*p* < 0.05, significant), ∗∗*p* < 0.01; ∗∗∗*p* < 0.001; ∗∗∗∗*p* < 0.0001. Individual *p* values are provided in Supplementary Table S4. The MICs of APR and AMK for the studied strain are 1 and 4 μg/mL, respectively.

To determine whether the encouraging efficacy of APR that we have previously observed in a hollow fibre infection model^30^ and an *M. abscessus* mouse infection model^11^ holds up against another bacterial strain and in a CF surrogate model, we infected CFTR^−/−^ mice by intratracheal inoculation with 0.5–1 × 10^6^ CFU of *M. abscessus* subsp. *abscessus* strain 4530, resulting in an average bacterial burden of 4.9–5.3 log CFU/g tissue at the start of treatment (SOT) two days post infection. In the vehicle treated control group (VC), bacterial burden increased to an average 5.5–6.0 log CFU/g lung, spleen, and liver tissue on day 12 post infection (Fig. 4).

Eight days of subcutaneous treatment with APR dose levels ≥64 mg/kg resulted in a 2-log CFU reduction relative to SOT in lung (*p* < 0.0001) and a statistically significant 1-log CFU reduction in spleen and liver. Although the log CFU reduction at 64 mg/kg was consistently higher for APR than for AMK, the difference was not statistically significant. CFU reduction in spleen and liver was not statistically significant for 64 mg/kg of AMK (Fig. 4, Supplementary Table S4). A dose level of 16 mg/kg APR resulted in only a small CFU reduction when compared to SOT, but in a statistically significant lower bacterial burden when compared to the VC. An identical dose of AMK did not result in a statistically significant CFU reduction relative to SOT or VC.

## Discussion

This study assessed multiple preclinical parameters of the clinical-stage drug candidate APR, contributing to a comprehensive evaluation for its therapeutic potential in NTM infections. Direct comparison to AMK, a current standard of care aminoglycoside antibiotic in the treatment of NTM infections, provided a preclinical reference point during assessment. The finding that APR is eightfold more active than AMK in inhibiting *M. abscessus*, consistent across several hundred contemporary clinical isolates of diverse geographic origin and across different testing sites, is particularly encouraging in view of the very narrow therapeutic window of AMK. In the context of previous reports on the clinical pharmacokinetics and safety of APR available to date, the higher in-vitro activity demonstrated in the present study may ultimately translate into a significantly wider therapeutic window in the treatment of *M. abscessus* infections when compared to the standard of care drug AMK. Although well-established pharmacodynamic animal models for *M. abscessus* infections are lacking, further work may be required to provide additional data sets in support of PKPD modelling of exposure targets and dose prediction.

The MIC distribution of APR for 358 *M. abscessus* isolates suggested an MIC_50_ of 2 μg/mL, an MIC_90_ of 4 μg/mL, and a 99% TECOFF of 4 μg/mL when applying the CLSI broth microdilution methodology, which suggests incubation at 30 °C to support growth of NTM species that grow less well at higher temperatures. This relatively low incubation temperature makes a lot of sense from the perspective of a diagnostic laboratory seeking to standardise its protocol across NTM species. However, our results indicate that the MICs of APR and AMK determined for *M. abscessus* at 30 °C may underestimate the antibacterial activity at 35 °C by about fourfold. This may be an important consideration during future PKPD modelling when we assume a temperature of 35 °C more closely resembles the temperature at the sites of infection.

Such a pronounced effect of the incubation conditions on MIC also calls for caution when comparing susceptibility results between sites (Supplementary Fig. S8) and between results reported in the literature. We considered it important to test the comparator AMK side by side with APR in the same assay plates. The MIC_50_, MIC_90_, and 99% TECOFF of AMK were eightfold higher than for APR, and resembled the AMK ECOFF of 64 μg/mL recently reported by twelve European laboratories.^31^ The CLSI clinical breakpoint of AMK has been set to 16 μg/mL for *M. abscessus*, which may cover its MIC_50_ but certainly not its MIC_90_ or ECOFF, whereas a hypothetical clinical breakpoint of 16 μg/mL for APR would be fourfold higher than its ECOFF. Although it may seem tempting to attribute frequent treatment failures of AMK to its MIC distribution extending well beyond the established clinical breakpoint, one needs to keep in mind that the AMK MICs may as well be lower at physiologic temperatures at the sites of infection than determined at 30 °C.

Although the MIC distributions appeared unimodal overall, the presence of a very few isolates with high-level resistance to both AMK and APR made us wonder about the rate of spontaneous resistance. The number of *M. abscessus* isolates with high-level resistance to AMK was low in the multicentre panel of isolates tested. This may arguably be surprising considering that long-term AMK is an integral part of many treatment regimens for *M. abscessus*, both as an injectable and as inhalation. For isolates with an AMK MIC of ≥128 μg/mL, APR did not provide any benefit over AMK, indicating that the mechanism of resistance may be due to target site alteration by mutations in ribosomal RNA, a mechanism that has been described to be the predominant clinical determinant of high-level AMK resistance.^32^^,^^33^

The frequency of spontaneous mycobacterial aminoglycoside resistance determined in this study appears to be considerably higher than reported previously for Gram-negative pathogens,^34^ but is in line with previous reports on mycobacterial aminoglycoside resistance by means of mutations in the 16S rRNA gene,^35^ which, unlike for many other bacterial species, is encoded by only a single gene copy in *M. abscessus*.^33^ The relatively high frequency of resistance in culture flask experiments may add to the surprise around the relatively low prevalence of AMK resistance that we observed in the large panel of clinical isolates tested. This may possibly be due to a fitness cost associated with mutations in ribosomal RNA. But since studying the mechanism and cost of resistance to AMK and APR was beyond the scope of the present work, further studies will be required to more closely assess the propensity for emergence of APR resistance in a clinical setting.

For NTM species other than *M. abscessus*, the APR MICs were four-to eight-fold lower than the AMK MICs for *M. chelonae* as well. However, for MAC, *M. fortuitum, M. kansasii M. xenopi,* the MIC distributions of APR and AMK were essentially superimposable. This inevitably raised the question of the molecular mechanism behind the superior activity of APR against *M. abscessus* and *M. chelonae*, but none of the other species tested. Although more detailed studies may be advisable, the findings appear to be consistent with previous studies showing that *M. abscessus* and *M. chelonae* possess the *eis2* gene encoding a promiscuous multi-acetyltransferase that confers resistance to AMK and other aminoglycosides but spares APR.^11^^,^^36^ The higher bactericidal activity of APR when compared with AMK has been attributed to the Eis2 multi-acetyltransferase as well.^11^ Meanwhile, *M. fortuitum* and SGM species have not been reported to have an *eis2* gene, likely explaining their equipotent APR and AMK MIC distributions. The Tsodikova group has previously reported on the taxonomic distribution of *eis* genes.^37^ Our own contemporary BLAST search against the Eis2 protein present in ATCC 19977 (WP_005079811.1) confirmed the presence of proteins with an amino acid sequence homology of greater than 84% homology in the genomes of the three *M. abscessus* subspecies and *M. chelonae*. Whereas less than 35% sequence homology was found across all MAC species, *M. fortuitum*, *M. kansasii*, and *M. xenopi* (Supplementary Table S5).

Determining MICs is a scalable methodology to assess drug susceptibility of larger number of bacterial isolates. However, to differentiate between a merely bacteriostatic growth inhibition and bactericidal killing, and to translate test tube MICs into a predicted efficacy at the site of infections, additional preclinical assessments are required.

We used a preclinical PKPD-qualified CFU count assay to confirm the more rapid killing of *M. abscessus* by APR than by AMK that we have demonstrated previously using a different methodology.^11^^,^^30^ The finding that the difference in bactericidal potency between APR and AMK is even greater at acidic pH is in agreement with a similar observation previously reported for Gram-negative pathogens, and of interest in cases where the site of infection is assumed to be a slightly acidic environment as well.^14^

APR activity was not compromised in the presence of sputum and killed stationary-phase *M. abscessus* in the presence of caseum surrogate. Exposure targets required for bacterial killing of *M. abscessus* in caseum surrogate have been demonstrated to be achievable for AMK, but not for most other NTM drugs including clarithromycin, linezolid, bedaquiline, and clofazimine.^24^ It was therefore encouraging to see that APR killed *M. abscessus* more effectively than AMK in the presence of caseum surrogate as well.

Intracellular killing of *M. abscessus* by APR inside macrophages may seemingly contradict a widespread perception of polar aminoglycosides being poor penetrators of eukaryotic cells. However, our finding is in agreement with our recent report on bactericidal activity of APR (but not of AMK) on intramacrophage *M. tuberculosis*.^25^ The same study has also demonstrated APR activity against AMK-resistant strains of *M. tuberculosis*, as well as biofilms, and efficacy of APR in a *M. tuberculosis* pulmonary mouse infection model, demonstrating both monotherapeutic efficacy and additive efficacy when combined with standard of care regimen, isoniazid, rifampicin, pyrazinamide, and ethambutol (HRZE).^25^ Another indication for the ability of APR to enter macrophages is the accumulation of APR in the human alveolar macrophages of healthy volunteers that received APR by intravenous infusion, as it has recently been reported online for clinical trial ID NCT05590728 (manuscript in preparation).

Considering the lung exposure and drug concentration in mouse epithelial lining fluid (ELF) following systemic administration of APR,^16^^,^^17^ it may not be surprising that the *in vitro* activity of APR has translated to mycobacterial CFU reduction in mouse lungs.^11^^,^^25^ However, uncertainty had remained about the fraction unbound in the presence of ELF, mucus, or even sputum, and whether the pulmonary efficacy would perhaps be compromised in such conditions that more closely resemble the conditions one would expect in patients generally and in patients with CF more specifically. It was therefore reassuring to find that the *in vitro* activity of APR was not hampered by patient sputum, and that the dose-dependent CFU reduction of *M. abscessus* in CF mice in response to APR treatment resembled the dose-dependent efficacy reported previously for SCID mice.^11^

Collectively, our studies on APR in *M. tuberculosis* and *M. abscessus* show a good *in vitro* to *in vivo* translation, which is a recurring challenge in the discovery and development of new antimycobacterials.^6^ However, this study also has its limitations. Our susceptibility testing included other commonly used comparator antimicrobials against NTM, such as clarithromycin, for only a subset of all isolates tested to better characterise and compare the isolates. Furthermore, colony morphology was not recorded by all sites, and results could thus be stratified by this variable for a subset of isolates only. Although animal models for NTM infections are still not ideal when compared to animal models for infections with other bacterial pathogens,^38^ conducting additional *in vivo* studies will nevertheless be instrumental in corroborating our collective results reported here and previously. Generating dose–response curves for additional isolates of different MICs, for instance, and for both male and female mice, will allow for a more robust pharmacodynamic assessment to inform the exposure targets during clinical development. Similarly, it will be essential to understand the added value of APR in the therapy of infected animals when combined with standard of care drugs, similar to how the authors have recently reported for *M. tuberculosis*.^25^

In conclusion, the APR MIC_90_ was four-to eight-fold lower than that of AMK against *M. abscessus* and *M. chelonae*. APR exhibited more potent and more rapid planktonic and intracellular killing, less frequent spontaneous resistance development than AMK, and uncompromised inhibition in sputum and caseum surrogate against *M. abscessus*. This *in vitro* and intramacrophage activity translated to *in vivo* efficacy in a CFTR^−/−^ mouse model of *M. abscessus* infection. Thus, APR is a potent aminoglycoside against NTM, especially against *M. abscessus*, and warrants further investigation. Previous PKPD studies suggested exposure targets required to cover an MIC_90_ of 8–16 μg/mL for Gram-negative pathogens. A more holistic data package including dose-fractionation PD for several *M. abscessus* strains would be required for a meaningful PKPD assessment for NTM infections. While APR has already undergone extensive preclinical profiling for the treatment of Gram-negative systemic infections, this study adds a comprehensive preclinical profiling towards assessing its potential in the treatment of mycobacterial infections.

## Contributors

Conceptualisation: SM, CLD, SNH. Data curation: MHN, MP, DV, MX, VC, KH, PK, SM, SNH. MHN and SNH have accessed and verified the underlying data. Formal analysis: DV, MX, KH, PK, SNH. Funding acquisition: KB, SM, CLD, SNH. Investigation: MHN, MP, DV, RVK, MX, VC, KH, RL, KB, PK. Methodology: DV, RVK, MX, KH, KB, RKS, TD, BS, SNH. Project administration: MP, VC, KH, KB, SM. Resources: TD, SN, BS, SM, CLD, SNH. Visualisation: MHN, DV, MX, KH, PK, RKS, SNH. Writing—original draft: MHN, SNH. Writing—review and editing: MHN, KH, PK, RKS, MS, ZPB, TD, BS, SM, CLD, SNH. All authors have read and approved the final version of the manuscript.

## Data sharing statement

All data reported in this study will be made available, to parties with legitimate interest, upon request to sven.hobbie@usb.ch.

## Declaration of interests

MHN has participated in a one-time advisory board meeting for Paratek Pharmaceuticals. CLD received research grants/contracts from AN2 Therapeutics, Bugworks, Insmed, Paratek, the Cystic Fibrosis Foundation, the COPD Foundation, Spero, Verona/Merck, Renovion, and MannKind. CLD has served on advisory boards for and as a consultant to AN2 Therapeutics, AstraZeneca, Insmed, Paratek, Juvabis, Galapogos, Grifols, Spero, GSK, Hyfe, MannKind, MicuRx, and NobHill. CLD has been a member of Data Monitoring Committees for Otsuka and the Bill and Melinda Gates Foundation. CLD is a shareholder in Nobhill. ZPB has received investigator-initiated research grants from Merck. All other authors declare no conflict of interests.
