# Supplementary material for: Multicentre preclinical profiling of apramycin for the treatment of nontuberculous mycobacteria
Source: eBioMedicine. 2025 Dec 31;123:106103. doi: 10.1016/j.ebiom.2025.106103 (PMC12804157; doi:10.1016/j.ebiom.2025.106103)
Supplement: Supplementary Tables Figures [file mmc1.pdf]

Multicentre preclinical profiling of apramycin for the treatment of nontuberculous mycobacteria

Minh-Vu H. Nguyen, Michel Plattner, Deepshikha Verma, Ramya V. Krishnamurthy, Min Xie, Vinicius Calado Nogueira de Moura, Klara Haldimann, Ramon Lang, Katja Becker, Parvinder Kaur, Radha Krishan Shandil, Mayas Singh, Zackery P. Bulman, Thomas Dick, Shridhar Narayanan, Bettina Schulthess, Satoshi Mitarai, Charles L. Daley, Sven N. Hobbie

Supplementary Material

Supplementary Table S1 ..... S2

Supplementary Table S2 ..... S3

Supplementary Table S3 ..... S4

Supplementary Table S4 ..... S5

Methodological Details of Animal Infection Model S6

Supplementary Fig. S1 ..... S7

Supplementary Fig. S2 ..... S8

Supplementary Fig. S3 ..... S9

Supplementary Fig. S4 ..... S10

Supplementary Fig. S5 ..... S11

Supplementary Fig. S6 ..... S12

Supplementary Fig. S7 ..... S14

Methodological Details of Intracellular Killing Assay S15

Supplementary Fig. S8 ..... S16

Supplementary Table S5 ..... S17

Supplementary Table S1. Total number of mycobacterial isolates tested, stratified by species, subspecies, and testing site.

	NJH	UZH	RIT	Total
**RGM**				
M. abscessus subsp. *abscessus*	54	56	60	170
M. abscessus subsp. *massiliense*	52	33	60	145
M. abscessus subsp. *bolletii*	12	31	0	43
M. chelonae	9	11	5	25
M. fortuitum	13	10	21	44
Total number of RGM	**140**	**141**	**146**	**427**
**SGM**				
M. avium	46	44	63	153
M. intracellulare subsp. *intracellulare*	37	39	63	139
M. intracellulare subsp. *chimaera*	29	39	0	68
M. kansasii	2	9	20	31
M. xenopi	0	6	4	10
Total number of SGM	**114**	**137**	**150**	**401**
Total number of NTM isolates				**828**

NJH, National Jewish Health (Denver, CO, USA); UZH, University of Zurich (Zurich, Switzerland); RIT, Research Institute of Tuberculosis (Kiyose-shi, Tokyo, Japan); RGM, rapidly growing mycobacteria; SGM, slowly growing mycobacteria; NTM, nontuberculous mycobacteria.

Supplementary Table S2. Material and testing conditions used at each site

	NJH	UZH	RIT
**Mueller Hinton**			
Supplier/Brand	Sigma	Sigma	BD
Catalogue number	90922	70192	212322
Lot number	BCCF0168	BCCH5267	1089790
Cation adjustment	as supplied	$CaCl_2$ (Merck)<br>$MgSO_4$ (Fluka)	as supplied
**Middlebrook OADC**			
Concentration	SGM: 5%	SGM: 5%	SGM: 5%
Supplier/Brand	Self-made	BD	BD
Catalogue number	-	212240	211886
Lot number	20220702	1279188	2308030
**Preculture growth**	7H11 agar plates	7H10 agar plates	7H10 agar plates
**Inoculum size**	0.0025 McFarland	0.005 McFarland	10^5 CFU/mL
**QC strains**	*M. peregrinum* ATCC 700686<br>*M. marinum* ATCC 927	*M. peregrinum* ATCC 700686<br>*M. marinum* ATCC 927	*M. peregrinum* ATCC 700686<br>*M. marinum* ATCC 927
**Incubation conditions**			
Plate sealing	Covered with lid in Ziplock bags	Sealed<br>SGM: wrapped	Sealed<br>& wrapped
Atmosphere	Air	Air	Air
Temperature			
RGM	30 ± 2°C	30 ± 2°C	30 ± 2°C
SGM	35 ± 2°C	36 ± 2°C	36 ± 1°C
M. xenopi	42 ± 2°C	40 ± 2°C	36 ± 1°C
**Incubation time**			
RGM	3-5 days	2-5 days	3-5 days
SGM	7-14 days	7-14 days	7-14 days

Supplementary Table S3. Effect of incubation temperature and pH on the MIC of apramycin (APR) and amikacin (AMK)

	MIC (mg/L)		
	30°C, pH7.3	35 ± 2°C pH7.3	35 ± 2°C pH6.0
M. abscessus ATCC 19977			
APR	2	0.5	16
AMK	8-16	2	64-128

Supplementary Table S4. Bacterial CFU counts and statistical analysis results for the treatment groups of the *Cftr*$^{-/-}$ mouse lung infection model with an intratracheal inoculation of 0.5 - 1 × 10^{6} CFU of *M. abscessus* 4530 per mouse.

	Dose level (mg/kg)	Route	Frequ.	Daily dose (mg/kg)	Treatment duration	Mice (n)	Log CFU Lung	Spleen	Liver
Pretreatment	-	-	-	-	-	5	5.39 ± 0.13	4.93 ± 20.4	5.00 ± 0.25
Vehicle	0	SC	BID	0	8 days	5	5.78 ± 0.15	5.25 ± 0.12	5.68 ± 0.14
APR	4	SC	BID	8	8 days	5	5.78 ± 0.15	5.25 ± 0.12	5.22 ± 0.16
APR	16	SC	BID	32	8 days	5	3.94 ± 0.53	4.81 ± 0.34	3.87 ± 0.03
APR	64	SC	BID	128	8 days	5	3.18 ± 0.26	3.42 ± 0.24	3.91 ± 0.27
APR	256	SC	BID	512	8 days	5	2.56 ± 0.21	3.82 ± 0.15	3.78 ± 0.28
AMK	16	SC	BID	32	8 days	5	5.56 ± 0.21	5.11 ± 0.14	5.27 ± 0.15
AMK	64	SC	BID	128	8 days	5	3.82 ± 0.24	4.30 ± 0.10	4.72 ± 0.14

p values of one-way ANOVA multiple comparisons of log CFU

		LUNG							
		SOT	VEH	APR 4	APR 16	APR 64	APR 256	AMK 16	AMK 64
SPLEEN	SOT		0.0697	0.7871	0.9947	<0.0001	<0.0001	0.2410	<0.0001
	VC	0.1129		0.5173	0.0219	<0.0001	<0.0001	0.9841	<0.0001
	APR 4	0.3074	0.9930		0.4453	<0.0001	<0.0001	0.9138	<0.0001
	APR 16	0.3745	0.0016	0.0067		<0.0001	<0.0001	0.0911	0.0002
	APR 64	0.0045	<0.0001	<0.0001	0.2365		0.5227	<0.0001	0.1760
	APR 256	0.0002	<0.0001	<0.0001	0.0219	0.7888		<0.0001	0.0055
	AMK 16	0.4629	0.9420	0.9996	0.0134	<0.0001	<0.0001		<0.0001
	AMK 64	0.4090	0.0018	0.0078	>0.9999	0.2129	0.0190	0.0155	
LIVER	SOT								
	VC	0.0331							
	APR 4	0.4966	0.5824						
	APR 16	0.9117	0.0035	0.1063					
	APR 64	0.0234	<0.0001	0.0004	0.1639				
	APR 256	0.0053	<0.0001	<0.0001	0.0477	0.9893			
	AMK 16	0.5915	0.4878	0.9998	0.1405	0.0006	0.0001		
	AMK 64	0.9979	0.0126	0.2708	0.9949	0.0587	0.0146	0.3402	

Methodological Details of Animal Infection Model

1. 6-8 week old B6*Cftr*$^{tm1UNC/tm1UNC}$ female mice were bred at Colorado State University laboratory Animal Resources breeder facility. Mice are rested one week before infection.
2. The acute *Cftr*$^{-/-}$ mouse model received an intratracheal infection with 1×10^6 CFU/mouse (*M. abscessus subspecies abscessus* 4530).
3. Three mice were sacrificed on day 2 post-infection to determine bacterial uptake. Whole lungs, spleens, and livers were extracted, each homogenized in 4.5 mL of 1× PBS and serial tenfold dilutions plated on 7H11/OADC agar plates. The plates are placed in 32ºC dry-air incubator for ~7 days.

   Therapy began on day 2 post infection and dosing continued for eight consecutive days: saline plasmalyte control, APR 4 mg/kg subcutaneous injection BID, APR 16 mg/kg subcutaneous injection BID, 64 mg/kg subcutaneous injection BID, 256 mg/kg subcutaneous injection BID, AMK 16 mg/kg subcutaneous injection BID, and AMK 64 mg/kg subcutaneous injection BID. Mice were weighed and clinically scored daily. Each test group consisted of 5 mice. Mice were sacrificed two days after administering the last dose of test article (day 10 post-infection). Five mice from each group were sacrificed, and bacterial loads determined by plating serial tenfold dilutions of lung, spleen, and liver homogenates.
4. Statistical analysis was performed by first converting CFU to logarithms, which were evaluated by a one-way ANOVA followed by a Dunnett's Multiple Comparison Test. Normality assumption was confirmed by linearity in Q-Q plotting.

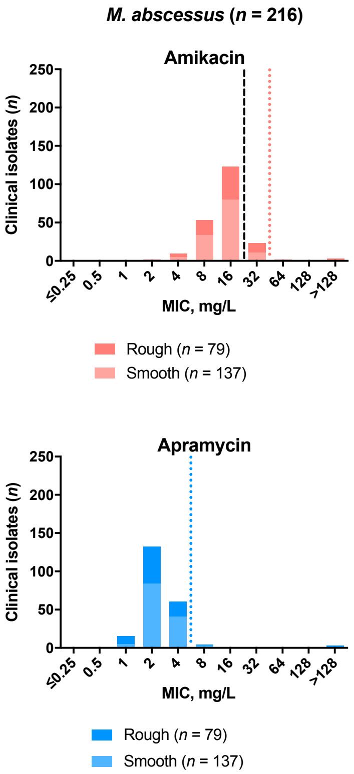

Supplementary Fig. S1. Minimal inhibitory concentration (MIC) distributions of apramycin and amikacin for *M. abscessus* isolates of smooth and rough morphotype. The tentative epidemiological cutoffs are indicated by a vertical dotted line. The black dashed vertical line indicates the Clinical and Laboratory Standards Institute breakpoint for amikacin (M24S, 2nd edition). Information on morphotype was only available for 216 of the 358 *M. abscessus* isolates tested overall.

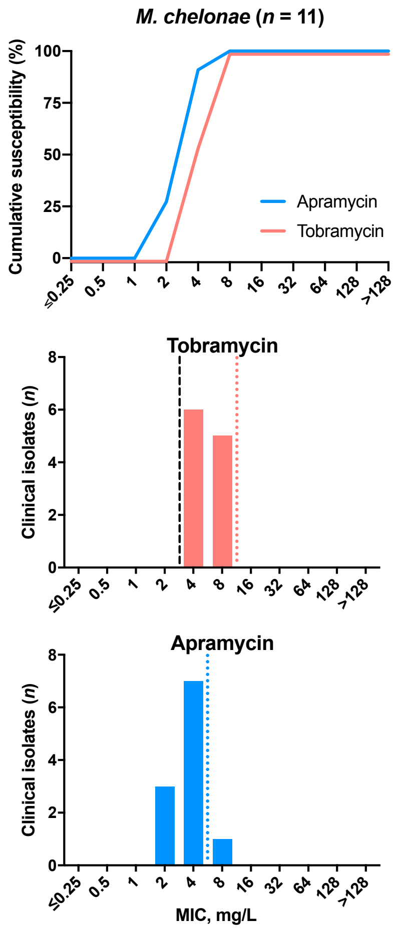

Supplementary Fig. S2. Cumulative susceptibility and minimal inhibitory concentration (MIC) distributions of tobramycin for the rapidly growing *M. chelonae*. Apramycin is shown for comparison. The tentative epidemiological cutoffs are indicated by a vertical dotted line. The black dashed vertical line indicates the Clinical and Laboratory Standards Institute breakpoint for tobramycin (M24S, 2nd edition).

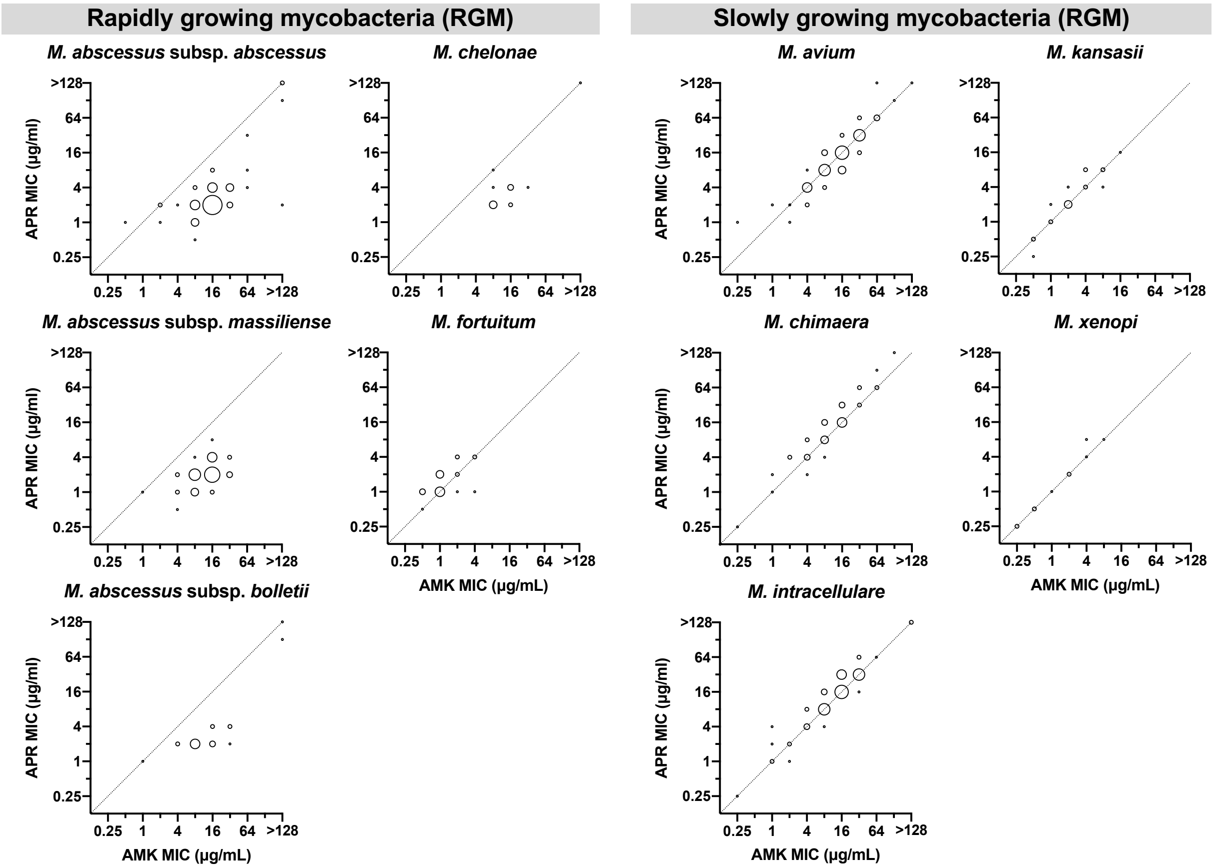

Supplementary Fig. S3. Bubble plot of the minimal inhibitory concentration (MIC) of apramycin (APR) plotted against the MIC of amikacin (AMK). Equipotency of identical MICs is indicated by a diagonal dotted line.

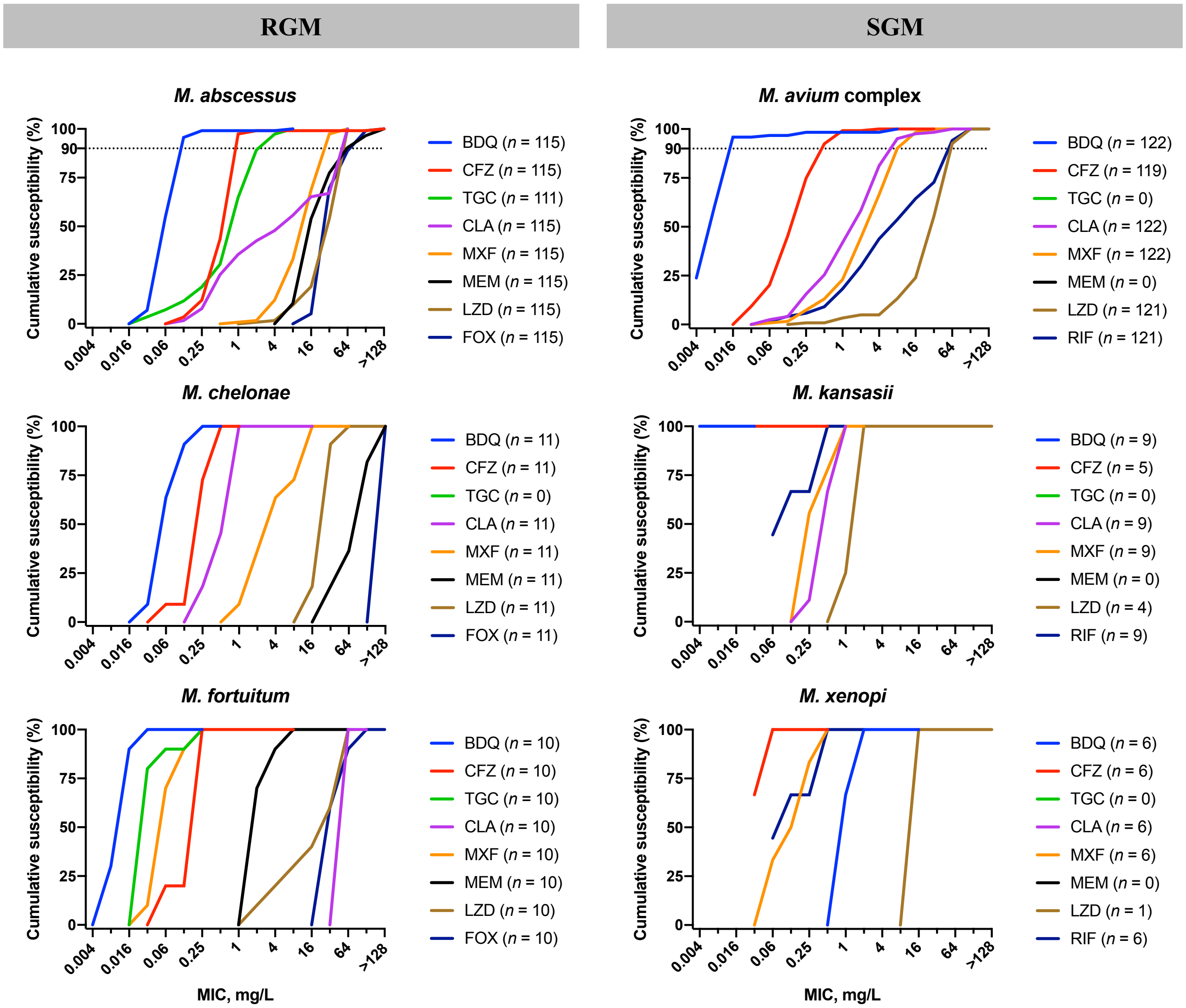

Supplementary Fig. S4. Cumulative susceptibility of a subset of European isolates to comparator drugs. The MIC_{90} cutoffs for *M. abscessus* and MAC isolates are indicated by a horizontal dotted line. RGM, rapidly growing mycobacteria; SGM, slowly growing bacteria. BDQ, bedaquiline; CFZ, clofazimine; TGC, tigecycline; CLA, clarithromycin; MXF, moxifloxacin; MEM, meropenem; LZD, linezolid; FOX, cefoxitin; RIF, rifampicin.

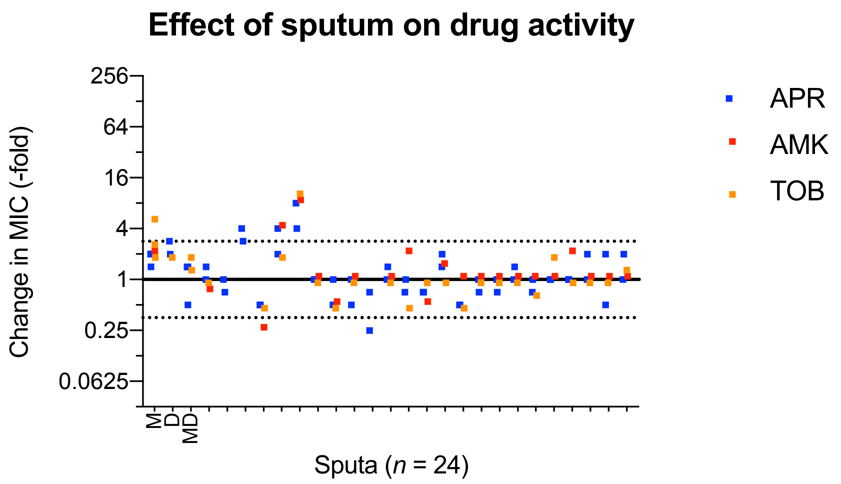

Supplementary Fig. S5. Sputum inhibition assays. M, 12% mucin control; D, 8 mg/mL calf thymus DNA control; MD, synthetic sputum surrogate comprised of a mixture of 12% mucin with 8 mg/mL DNA. The horizontal solid line corresponds to unaffected susceptibility (one-fold change in MIC). Horizontal dotted lines indicate an error margin of ± 1 × log_2 titer deviation.

CLARITHROMYCIN

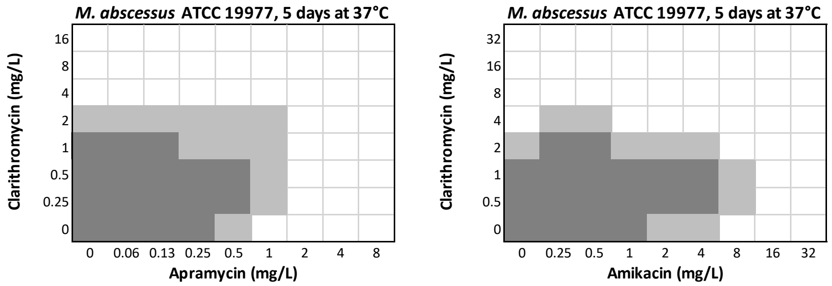

CLOFAZIMINE

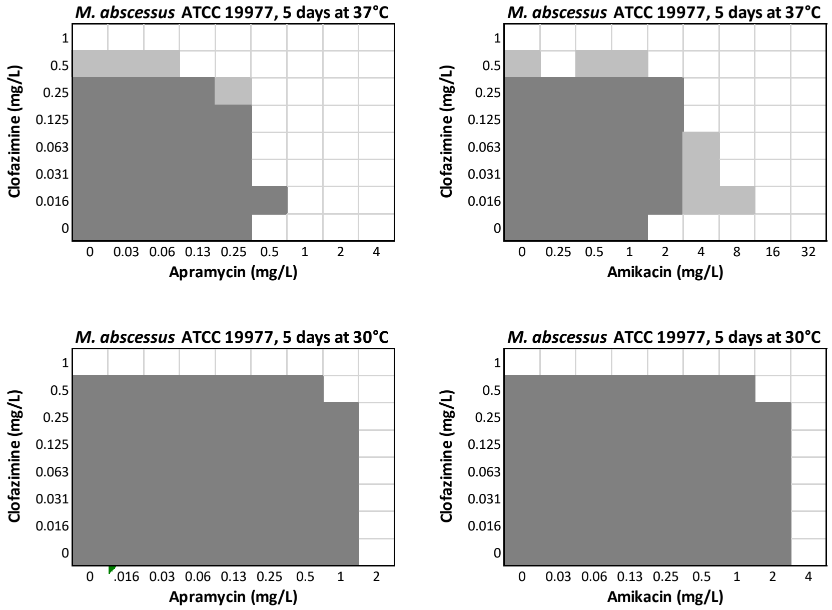

IMIPENEM

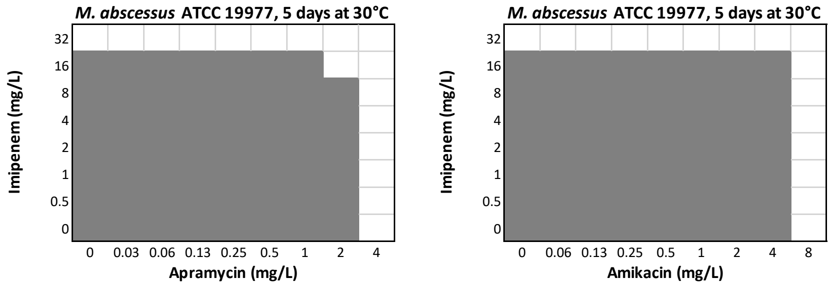

Supplementary Fig. S6. Antibacterial checkerboard assays with apramycin and amikacin.

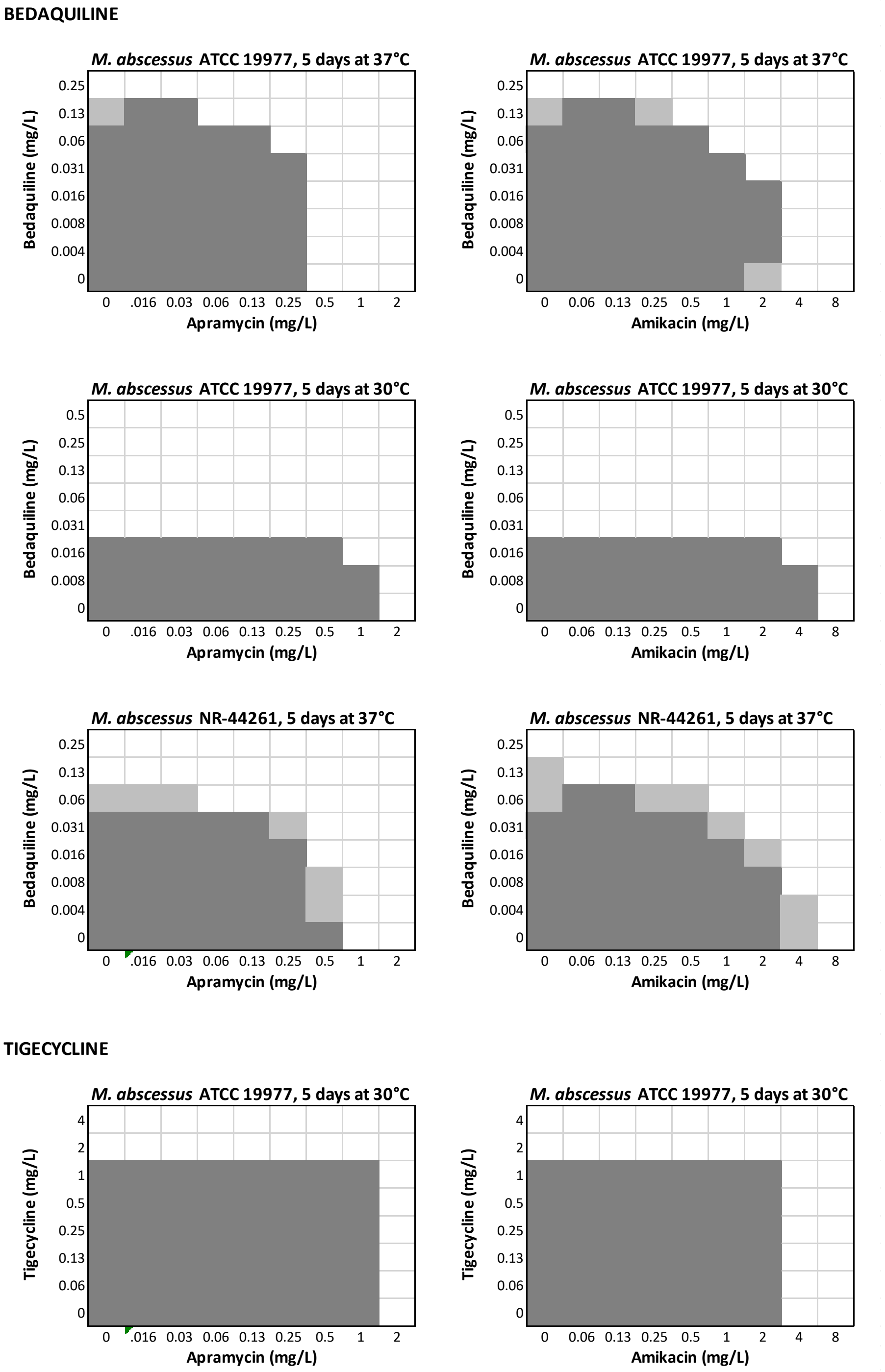

Supplementary Fig. S6 (cont'd). Antibacterial checkerboard assays with apramycin and amikacin.

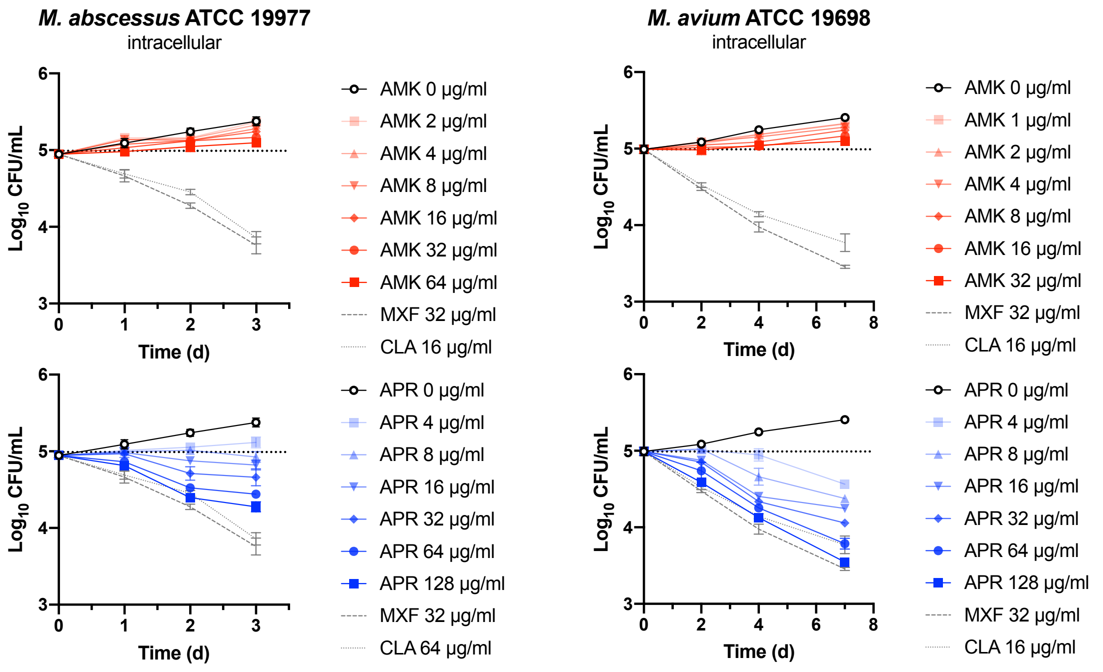

Supplementary Fig. S7. Time resolution of intracellular killing of *M. abscessus* ATCC 19977 and of *M. avium* ATCC 19698 inside THP-1 macrophages by APR and AMK in comparison to moxifloxacin (MXF) and clarithromycin (CLA), data plotted as mean ± SD; *n* = 3 biological replicates. Dose response curves for day 3 and day 7, respectively, are plotted in Fig. 3.

Methodological Details of Intracellular Killing Assay

1. THP-1 cells were seeded at a density of 5×10^5 cells/well into RPMI medium (Gibco) supplemented with 100 mM sodium pyruvate, 200 mM L-glutamine, 3.7 g/L sodium bicarbonate and 10% fetal calf serum without any antimicrobials. The viable THP-1 cells were seeded in 96-well plates (Nunc, Roskilde, Denmark) with complete RPMI at a density of approximately 5×10^5 cells/well and incubated overnight.
2. Macrophage differentiation was induced by 50 nM phorbol 12-myristate 13-acetate (PMA) and incubated at 37 °C/48–72 h/5% CO_2 atmosphere.
3. THP-1 macrophages were infected with *M. abscessus* ATCC 19977 or *M. avium* ATCC 19698 at a multiplicity of infection (MOI) of 1:10 and incubated for 2 h at 37°C with 5% CO_2. The monolayers in the wells were washed with phosphate-buffered saline (Ca^{2+}, Mg^{2+}) pH 7.0, and treated with AMK (50 µg/mL final concentration) at 37°C for 2 h to kill and remove the extra-cellular bacteria if any released before lysis of macrophages. The wells were washed with PBS pH 7.0 and replenished with fresh complete RPMI medium. Cells were lysed (0.05% SDS) and the lysate CFU was enumerated to estimate the numbers of intracellular NTM 2 h post-infection.
4. The remaining wells were used for infection controls, or for the drug-exposure at 2 h post-infection. After the treatment was performed with 4 – 128 APR µg/mL vs. 1 – 32 µg/mL AMK, as well as the control drugs moxifloxacin (MOX; 32 µg/mL), and CLA (32 µg/mL for *M. abscessus* and 16 µg/mL for *M. avium*) at specific time points (Day 3 for *M. abscessus*, Day-7 for *M. avium*).
5. The cultures from the treatment wells and the infection control wells were released from macrophages by lysing with 100 µL of 0.04% SDS, and were plated on 7H11 agar plates for CFU enumeration.
6. Following respective incubations, the colony forming units (CFU) were enumerated for the residual intracellular NTM. The killing curves were generated by plotting the log_{10} cfu/mL against different drug concentrations. The assay was performed as biological triplicates. The data was analyzed and plotted using the software GraphPad Prism V-9.
7. Uninfected THP-1 macrophages were treated and incubated in exactly the same way as above to microscopically control for any potential drug effects on cell layer integrity and cellular attachment during 7 days of incubation.

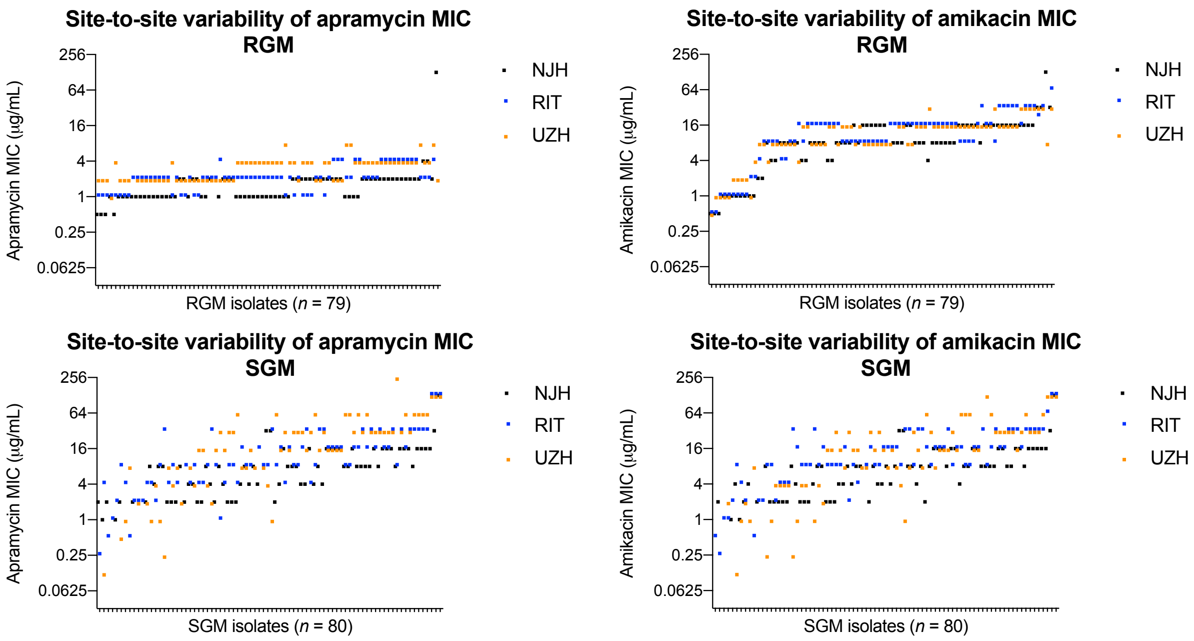

Supplementary Fig. S8. MIC variability between sites. Of the 828 isolates studied overall, 79 RGM and 80 SGM isolates were tested across all three sites (*n* = 3 independent replicates across three sites) to study variability of susceptibility results.

Supplementary Table S5. Amino acid sequence homologies to the *M. abscessus* ATCC 19977 Eis2 acetyltransferase. Based on a BLASTp homology search conducted on Oct 9th, 2024

Species	Subspecies	NCBI taxonomy ID	BLASTp residue identities Eis2
M. abscessus	ATCC 19977	#561007	100.0%
M. abscessus	*abscessus*	#1185650	99.5%
M. abscessus	*bolletii*	#319705	84.4%
M. abscessus	*massiliense*	#1198627	99.3%
M. chelonae	various	#1774	84.7%
M. chelonae	various	#1774	84.7%
M. fortuitum	*fortuitum*	#144549	33.1%
M. fortuitum	various	#1766	31.0%
M. fortuitum	*acetamidolyticum*	#144550	33.1%
M. fortuitum	*fortuitum*	#1214102	33.1%
M. avium		#243243	32.9%
M. avium	*hominissuis*	#439334	32.9%
M. avium	*hominissuis*	#1402966	no significant similarity found
M. avium	*avium*	#553481	32.6%
M. intracellulare	*intracellulare*	#1232724	no significant similarity found
M. intracellulare	*intracellulare*	#35617	no significant similarity found
M. intracellulare	*chimaera*	#222805	33.6%
M. kansasii		#557599	34.5%
M. xenopi		#1789	no significant similarity found
